# Perinatal Inflammation Results in Sex-Dependent Cardiac Dysfunction

**DOI:** 10.3390/jcdd11110346

**Published:** 2024-11-01

**Authors:** Leeann R. Pavlek, Kathryn M. Heyob, Nitya R. Jacob, Saichidroopi Korada, Zahra Khuhro, Aiman Q. Khan, Terri A. Shaffer, Sara Conroy, Markus Velten, Lynette K. Rogers

**Affiliations:** 1 Center for Perinatal Research, The Abigail Wexner Research Institute at Nationwide Children’s Hospital, Columbus, OH 43215, USA; kathryn.heyob@nationwidechildrens.org (K.M.H.); terri.shaffer@nationwidechildrens.org (T.A.S.); sara.conroy@nationwidechildrens.org (S.C.); rogers.377@osu.edu (L.K.R.); 2 Department of Pediatrics, The Ohio State University, Columbus, OH 43215, USA; 3 Biostatistics Resource at Nationwide Children’s Hospital, Columbus, OH 43215, USA; 4 Center for Biostatistics, The Ohio State University Wexner Medical Center, Columbus, OH 43215, USA; 5 Department of Anesthesiology and Pain Management, University of Texas Southwestern Medical Center, Dallas, TX 75390, USA; markus.velten@utsouthwestern.edu

**Keywords:** growth restriction, cardiac dysfunction, sex differences

## Abstract

Background: An increased incidence of adult-onset heart failure is seen in individuals born preterm or affected by fetal growth restriction. An adverse maternal environment is associated with both preterm birth and poor fetal development, and postnatal oxygen therapy is frequently required to sustain oxygenation of vulnerable tissues due to lung immaturity. Methods: Studies using our murine model of maternal inflammation (LPS) and neonatal hyperoxia exposure (O_2_) observed pathological changes in cardiac structural proteins and functional analysis with sex dependent differences in pathologies at 10 months of age. Using our previous model, the current investigations tested the hypothesis that early-life perturbations in cardiac structural proteins might predict adult cardiac dysfunction in a sex dependent manner. Results: LPS-exposed females had lower αMHC mRNA and protein at P0 and P7 relative to the saline-exposed females, but these changes did not persist. Male mice exposed to LPS/O_2_ had normal expression of αMHC mRNA and protein compared to saline/room air controls though P56, when they dramatically increased. Correlative changes were observed in left ventricular function with a more severe phenotype in the males indicating sex-based differences in cardiac adaptation. Conclusions: Our findings demonstrate that early changes in contractile proteins temporally correlate with deficits in cardiac contractility, with a more severe phenotype in males. Our data suggest that similar findings in humans may predict risk for disease in growth-restricted infants.

## 1. Introduction

Adverse exposures during crucial windows in development, specifically throughout fetal and early postnatal life, can alter molecular signatures, resulting in impaired functions that affect health for the entire lifetime [1]. Many adult cardiovascular diseases have now been linked to fetal programming via epigenetic changes that affect cardiovascular remodeling and strengthen the link between the fetus and later adult disease; however, the role of sex in these mechanisms is largely unknown [2,3]. Although the heart is the first functioning organ to develop in the fetus [4], many molecular structures and advanced functions are still developing in the context of extraordinary plasticity at the time of birth. The transition to postnatal life is also associated with a switch from a cardiac hyperplastic to a hypertrophic pattern of growth [5]. Hostile perinatal environments can pathologically alter or interrupt these highly regulated developmental processes and result in long-term cardiac morbidities.

Preterm delivery at less than 37-week gestation affects approximately 10% of live births annually, and, of these, 5–12% experience intrauterine growth restriction and are born with low birth weights. [6]. More than half of preterm births are due to spontaneous preterm labor, which is often triggered by an intrauterine infection or inflammation [7]. These affected neonates often require respiratory support and supplemental oxygen to sustain tissue oxygenation due to immature lung development, which leads to oxidative stress in addition to prenatal exposure to inflammation. The role of inflammation and oxidative stress has been well documented in models of other morbidities associated with adverse birth environments, including bronchopulmonary dysplasia, retinopathy of prematurity, intraventricular hemorrhage, impaired myelination, and necrotizing enterocolitis [8,9,10].

For this investigation, we employed our well-established mouse model of perinatal inflammation (maternal inflammation (LPS) and neonatal hyperoxia exposure) [11,12,13]. This ‘dual-hit’ model mimics infants with severe prenatal and postnatal morbidity, including fetal growth restriction. We have previously observed pathological changes in cardiac structural proteins and functional analysis with sex dependent differences in pathologies at 10 months of age [11]. The goal of the present study was to systematically analyze growth-restricted mice exposed to our perinatal inflammation protocol to determine a time course for perturbations in structural proteins and how early changes in these proteins might predict adult cardiac dysfunction in a sex dependent manner.

## 2. Materials and Methods

### 2.1. Animal Model

All animals were handled in accordance with the National Institutes of Health guidelines. All protocols were approved by the Institutional Animal Care and Use Committee of the Abigail Wexner Research Institute at Nationwide Children’s Hospital (Columbus, OH, USA, IACUC # AR07-0028). Male and female C3H/HeN mice were paired and the presence of a vaginal plug was designated as embryonic day 1 (E1). On E16, dams were injected with lipopolysaccharide (LPS, 80 ug/kg intraperitoneal, serotype 0111:B4 Calbiochem, #437627), or an equal volume of saline (Sal).

On postnatal day 0 (P0), newborn mice were euthanized using ketamine and xylazine and whole-heart samples were collected. The two exposure groups at P0 were designated Sal (dam injected with saline) and LPS (dam injected with LPS). For all other time points, newborn mice from saline- or LPS-treated dams were placed in a plexiglass chamber containing 10 mL/minute flow of 85% oxygen for 14 days with a corresponding dam and litter maintained in room air. Subsequently all mice were raised in room air until the designated time points. To avoid oxygen toxicity in the dams and eliminate maternal effects between the two groups, the nursing dams were rotated between hyperoxia (O_2_) and room air (RA) litters every 24 h and the litters with the same intrauterine treatment were mixed. Twenty-four hours of hyperoxia exposure was designated as postnatal (P)1. The mice were euthanized after exposure to 85% oxygen or room air on P7 and after transfer to room air on day 14 at P21, or P56. Body and heart weights were recorded at the time of euthanasia. The resulting exposure groups were designated Sal/RA (maternal saline, postnatal room air), Sal/O_2_ (maternal saline, postnatal hyperoxia), LPS/RA (maternal LPS, postnatal room air), and LPS/O_2_ (maternal LPS, postnatal hyperoxia).

### 2.2. RNA Isolation and Quantitative Real-Time PCR

An RNeasy Mini kit (74104, Qiagen; Hilden, Germany) was used to isolate total RNA from whole-heart tissues at P0, P7, P21, and P56. cDNA was synthesized using a Maxima First Strand cDNA Synthesis Kit for RT-Quantitative PCR (K1642, Thermo Fisher; Waltham, MA, USA). A MasterCycler epgradient RealPlex RT-PCR Detection System (Eppendorf; Hamburg, Germany) was used for quantitative real-time PCR analyses with Maxima SYBR Green/ROX qPCR Master Mix (K0221, Thermo Fisher; Waltham, MA, USA). The primers used for qRT-PCR are listed in Table 1. mRNA samples were normalized to β-actin.

### 2.3. Western Blot

Whole-heart tissue homogenate proteins were separated on Tris-Acetate gels for myosin heavy chain 6 (MYH6 or alpha myosin heavy chain, αMHC) and myosin heavy chain 7 (MYH7 or beta myosin heavy chain, βMHC) using Tris-Acetate Running Buffer. After separation, the proteins were transferred onto PVDF membranes and were probed using the designated primary antibodies (MYH6, 1:200, sc-168676, Santa Cruz Biotechnology, Dallas, TX, USA), (MYH7, 1:200, sc-53089, Santa Cruz Biotechnology, Dallas, TX, USA). The membranes were subsequently probed with species-specific secondary antibodies (Bio-Rad, Hercules, CA, USA). The membranes were then developed using chemiluminescence (Clarity Max Western ECL substrate, Bio-Rad, Hercules, CA, USA), and the bands were imaged using ChemiDoc Imaging System (Bio-Rad, Hercules, CA, USA) or by film. The bands were quantified using Image Lab software (version 6.0, Bio-Rad, Hercules, CA, USA) and normalized to the density of the corresponding a-tubulin blot.

### 2.4. Echocardiography

Serial echocardiography was performed on the same animal at P14, P21, P28, and P56 using a Fujifilm VisualSonics Vevo3100 (Toronto, ON, Canada). The mice were sedated with 1.5% isoflurane (delivered in 100% O_2_) and body temperature was maintained at 37 °C. The MX400 transducer probe (20–46 MHz, Axial Resolution 50 um) was positioned on the chest with pre-warmed ultrasound gel (Aquasonic, Parker Labs, Fairfield, NJ, USA) to acquire the parasternal, long and short axis orientation. Three loops were captured in B-Mode and M-Mode from each mouse and the data were averaged from 5 beats. Left ventricle (LV) dimensions in systole and diastole, as well as posterior wall thickness in systole and diastole, were assessed (Figure S1). The parameters were assessed in accordance with the guidelines for measuring cardiac physiology in mice [14] and the measurements were analyzed using the American Society for Echocardiography recommendations [15]. The operator and the person analyzing the data were blinded to group assignment. The Vevo Lab echocardiogram analysis software (version 5.6.1) was used to determine the heart rate (HR), stroke volume (SV), cardiac output (CO), ejection fraction (EF), and fractional shortening (FS), of each specimen at all time points. FS was calculated using the following equation: FS = [(LV dimension in diastole − LV dimension in systole)/left ventricular dimension in diastole] × 100%. EF was calculated using the following equation: EF = [(LV end-diastolic volume − LV end-systolic volume)/LV end-diastolic volume] × 100. CO was calculated by: CO = HR × SV. Representative M-mode images for P56 are presented in the Supplemental Materials. The numbers of animals for each group are as follows: Sal/RA, m = 4, f = 7; Sal/O_2_, m = 6, f = 4; LPS/RA, m = 6, f = 3; LPS/O_2_, m = 5, f = 4. The same pups were analyzed over the time span and the statistics reflected repeated measures, which increased the power of the analyses. The numbers were variable due to the sex of pups born with litters and the need to have all pups analyzed at the same time to eliminate differences.

### 2.5. Statistical Analysis

Data are expressed as means ± standard error of the mean (SEM). Statistical analyses were performed with GraphPad PRISM Windows version 8.0.0 (San Diego, CA, USA) using a two-way ANOVA and Tukey’s post hoc. Echocardiogram measurements were analyzed using a linear mixed model to account for correlated measures over time and interaction of day of life, treatment, and/or sex with R [16]. Likelihood ratio tests compared nested models. *p* < 0.05 was considered statistically significant. The results were analyzed for sex differences.

## 3. Results

### 3.1. Mouse Body and Heart Weights

At P0, P7, P21, and P56, the mice were euthanized, and their body weights were recorded (Table 2). At P0, LPS-exposed mice of both sexes had significantly lower body weights than the corresponding saline-exposed mice. At P7, mice in both LPS groups (LPS/RA, and LPS/O_2_) and of both sexes had significantly lower body weights than the respective Sal/RA mice. By P21, males in the LPS-exposed groups weighed more than the saline-exposed groups, indicating “catch-up” growth. Females in the LPS/RA group weighed more than the Sal/O_2_, but the effects were less severe than those of the males. At P56, the effects of exposure were less profound but there were differences between sexes at every time point.

### 3.2. Heart Rates

**Figure 1:**
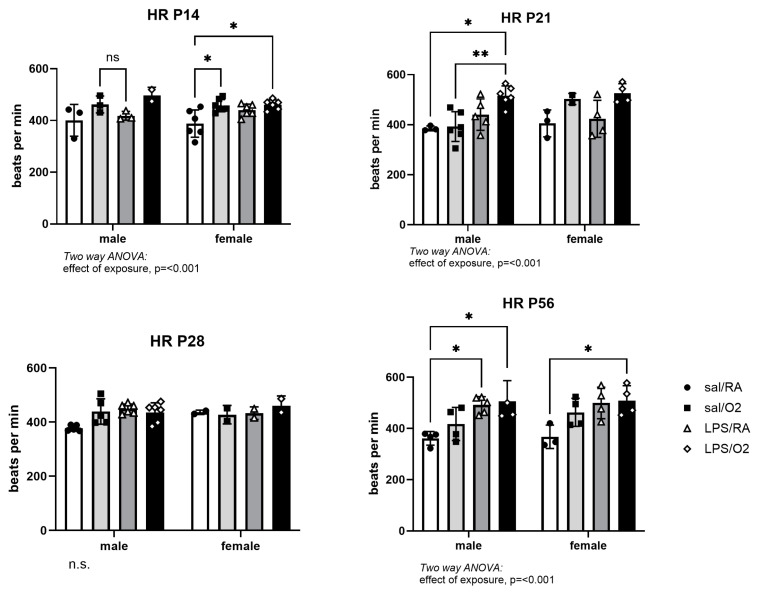
Heart rates were measured as a component of echocardiography. The data were analyzed by two-way ANOVA with Tukey’s post hoc. n = 3–6 pups from ≥3 litters per time point and treatment group. *p* < 0.05 was considered statistically significant; n.s., not significant; *, *p* < 0.05; **, *p* < 0.005.

Heart rates were measured as a function of echocardiography. There was a trend toward higher heart rates in the LPS-exposed mice at P14 and the males at P21 days (Figure 1). By P56, significantly higher heart rates were measured in the LPS/O_2_ exposed mice than Sal/RA controls in both sexes.

### 3.3. Echocardiography

**Figure 2:**
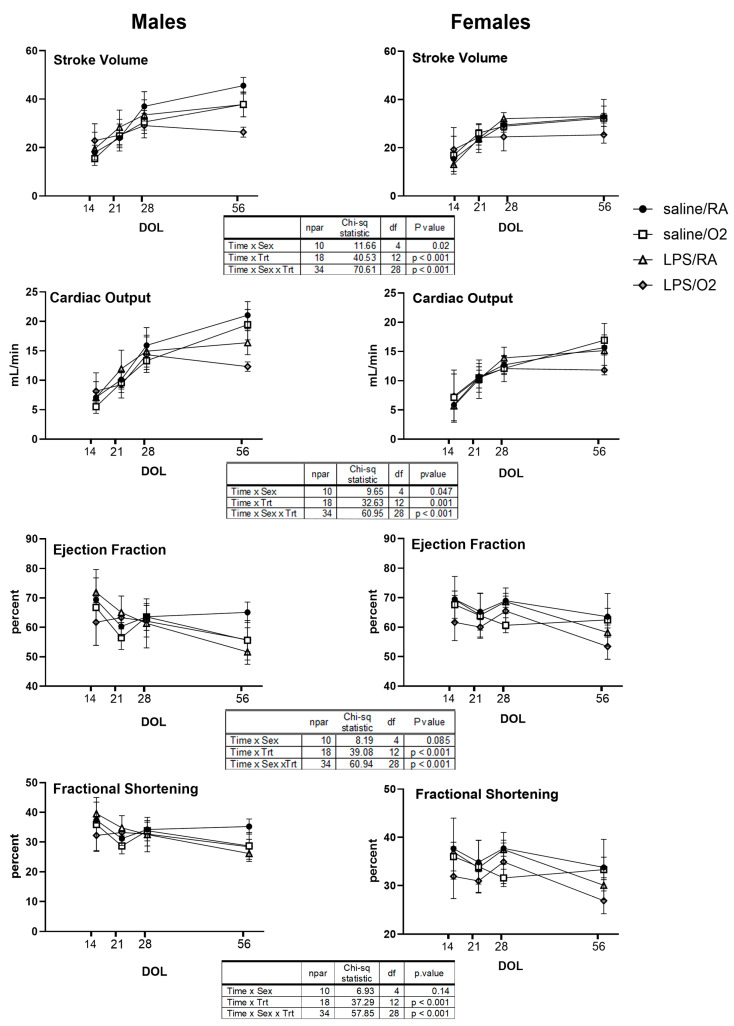
Serial echocardiography was performed on P14, P21, P28, and P56 using VisualSonics Vevo2100. Three loops were captured from each mouse and data were averaged from five beats. Repeated measure analyses were performed to identify differences between exposure groups and between sexes. Data were analyzed using a linear mixed model to correlate measures over time and interaction of day of life, treatment, and/or sex. The deviance from each model was compared for nested models with Chi-squared tests. *p* < 0.05 was considered statistically significant. The results were analyzed for sex differences. n = 3–6 (defined in methods) pups from ≥3 litters per treatment group.

Serial echocardiography was performed and estimated average values for the corresponding outcome across time and by sex from the linear mixed models are presented in Figure 2. At P14, for both sexes, the LPS/O_2_ groups had the lowest FS and EF. The FS and EF in the LPS-exposed groups in both sexes remained lower than in the Sal/RA groups through P56 (Figure 2). A modest improvement in FS and EF was seen between P21 and P28 for the LPS/O_2_ females and the Sal/O_2_ males, but a decline was seen again by P56.

**Figure 3:**
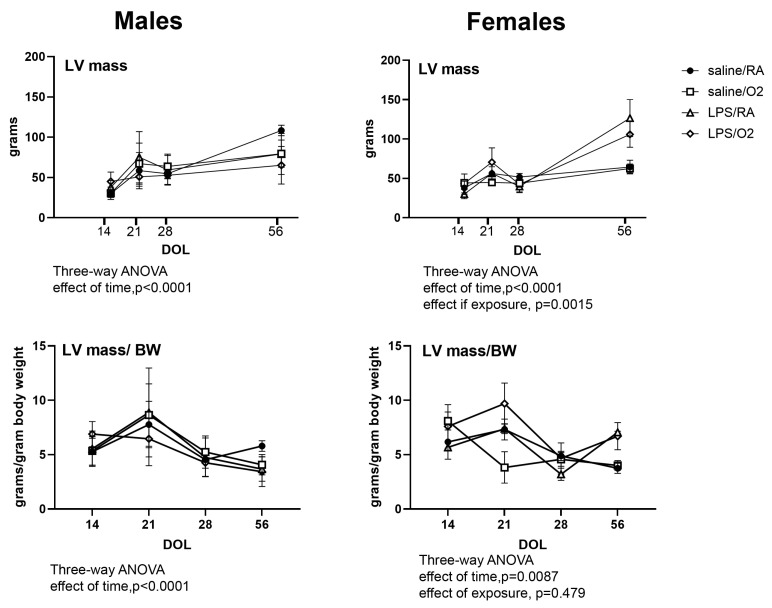
LV mass was obtained from serial echocardiography performed on P14, P21, P28, and P56. LV mass/body weight (BW) was calculated from the presented LV mass and the BW of each individual mouse and averaged for each group. Three loops were captured from each mouse and data were averaged from five beats. Repeated measure analyses were performed to identify differences between exposure groups and between sexes. Data were analyzed using repeated measures Three-way ANOVA, *p* < 0.05 was considered statistically significant. The results were analyzed for sex, time, and exposure differences. n = 3–6 pups (defined in Methods) from ≥3 litters per treatment group.

For both sexes, SV was highest in the LPS/O2 groups at P14 but lowest by P56. CO was also lowest in the LPS/O2 group at P56 with the male phenotype more severe. LV size over time was measured by the LV mass index (Figure 3) and LV volume (Tables S1 and S2). At P56 females had significantly higher LV mass index and at both P28 and P56 females had lower LV volumes in both LPS-exposed groups than the saline-exposed groups. For males by P56, all exposure groups had lower LV mass index and lower LV volumes than the Sal/RA control group. Normalizing LV mass to mouse body weight did not affect statistical significance (Figure 3). LV posterior wall thickness (LVPW) was also assessed, and no differences were observed in either sex or exposures (Table S3 and S4). 

### 3.4. MHC mRNA Expression

**Figure 4:**
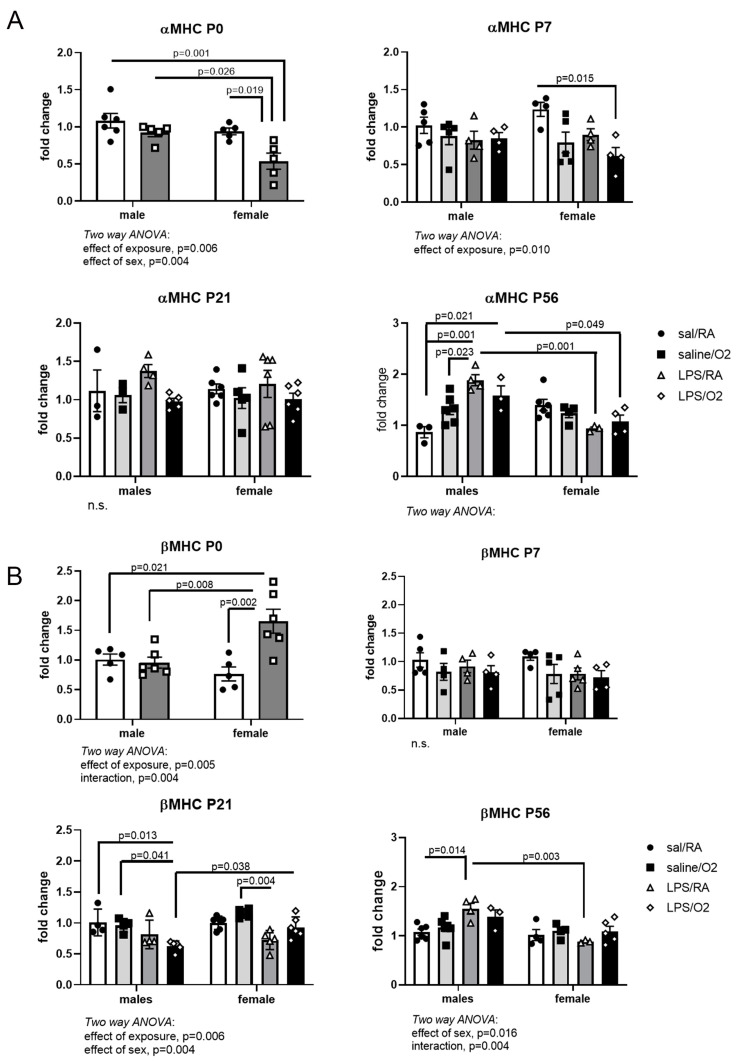
PCR analyses of MHC expression. αMHC (**A**) and βMHC (**B**) expressions were measured by RTPCR using standard techniques and β-actin as loading control. Each time point was normalized to Sal/RA males. Data were analyzed by two-way ANOVA with Tukey’s post hoc. The numbers of animals for each group are as follows; Sal/RA, m = 5, f = 6; Sal/O_2_, m = 4, f = 5; LPS/RA, m = 5, f = 4; LPS/O_2_, m = 5, f = 4 obtained from 3–4 litters per time point and treatment group (total = 15 litters), *p* < 0.05 was considered statistically significant; n.s., not significant.

αMHC and βMHC mRNA expression was measured by qRT-PCR in RNA isolated from whole-heart samples (Figure 4A,B). At P0, the female LPS-exposed group had lower expression of αMHC than all other groups. At P7, the female LPS/O_2_ group had lower expression than the female Sal/RA group. There were no differences noted at P21. At P56, males in both LPS-exposed groups had higher expression of αMHC than the Sal/RA group, while LPS-exposed females were not different than their respective Sal/RA group but had lower expression than similarly exposed males (Figure 4A).

Contrary to the effects on αMHC, at P0, the female LPS-exposed group had greater βMHC expression than all other groups. No differences in βMHC were observed at P7. At P21, the male LPS/O_2_-exposed group had lower βMHC expression than all other male groups and lower than female LPS/O_2_. At P56, LPS/RA exposed males had higher expression than their respective Sal/RA and the female LPS/RA groups (Figure 4B).

### 3.5. MHC Protein Levels

**Figure 5:**
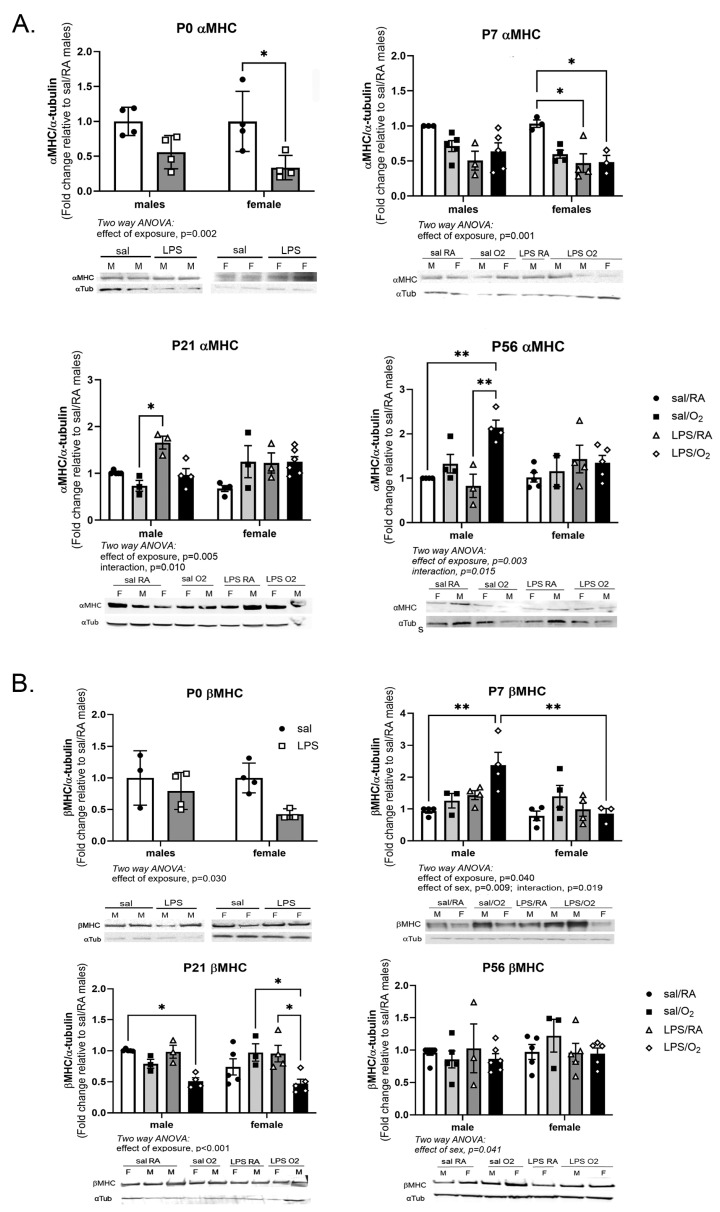
Western blot analysis of MHC protein. αMHC (**A**) and βMHC (**B**) levels were measured by standard Western blot techniques using α-tubulin as loading control. Each time point was normalized to Sal/RA males. Data were analyzed by two-way ANOVA with Tukey’s post hoc. The numbers of animals for each group are as follows; Sal/RA, m = 5, f = 6; Sal/O_2_, m = 4, f = 5; LPS/RA, m = 5, f = 4; LPS/O_2_, m = 5, f = 4 obtained from 3–4 litters per time point and treatment group (total = 15 litters). *p* < 0.05 was considered statistically significant; *, *p* < 0.05; **, *p* < 0.005.

αMHC and βMHC protein expression was measured by Western blot analysis (Figure 5A). At P0, LPS-exposed females had lower levels of αMHC than their respective saline controls. At P7, both female LPS-exposed groups had lower protein levels than the respective Sal/RA group. At P21, the male LPS/RA group had higher αMHC levels than the male Sal/O_2_ group. At P56, the levels remained higher in the male LPS/O_2_ group than in the male Sal/RA and LPS/RA exposure groups. Overall, exposed females tended to have lower levels, while exposed males tended to have higher levels of αMHC (Figure 5A).

At P0, there was an effect of exposure on βMHC but no post hoc differences were observed. At P7, the male LPS/O_2_ group had higher protein levels than the respective Sal/RA group or the female LPS/O_2_. At P21, both male and female LPS/O_2_ groups had lower βMHC levels than other groups of the same sex. By P56, there was an effect of sex, but no post hoc differences were identified (Figure 5B).

**Figure 6:**
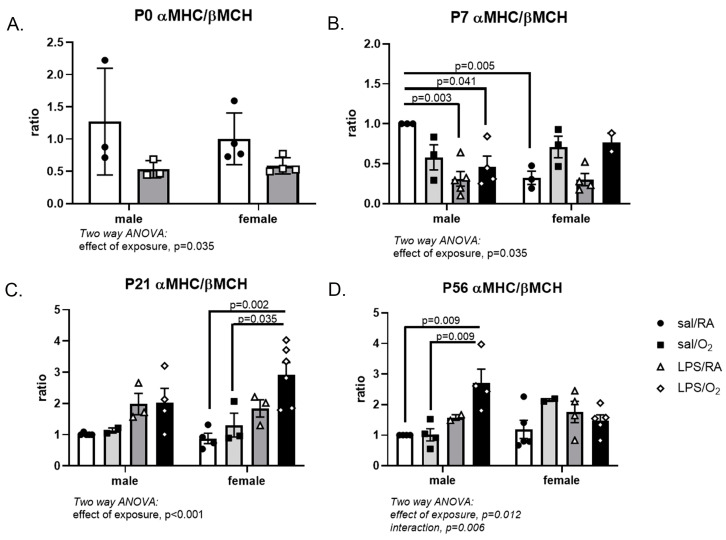
Ratio of αMHC and βMHC protein levels. Ratios were calculated from the levels of αMHC and βMHC protein obtained from the Western blots in Figure 4. Data were analyzed by two-way ANOVA with Tukey’s post hoc. n = 3–6 pups and 3–4 litters per time point and treatment group (total = 15 litters). *p* < 0.05 was considered statistically significant.

The ratios of αMHC to βMHC were calculated as indicators of cardiac function. At P0, the LPS-exposed groups had a lower trend than the saline-exposed groups. At P7, the male LPS-exposed groups had lower values than the Sal/RA control. Females had lower levels in the Sal/RA group than male Sal/RA. At P21, the female LPS/O_2_ group had much higher ratios than the female saline-exposed groups. By P56, these differences in females had resolved, but the male LPS/O_2_ group had a higher ratio than either of the male Sal-exposed groups (Figure 6).

### 3.6. Connexin-43 and Desmin

**Figure 7:**
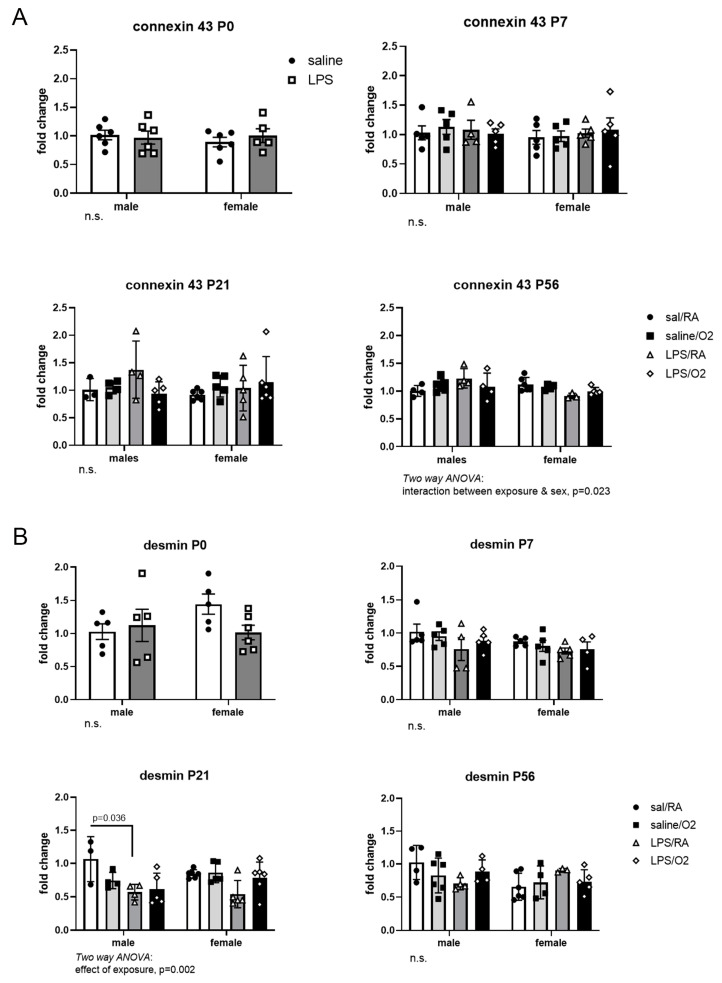
PCR analyses of connexin 43 and desmin expression. connexin 43 (**A**) and desmin (**B**) expressions were measured by RTPCR using standard techniques and β-actin as loading control. Each time point was normalized to Sal/RA males. Data were analyzed by two-way ANOVA with Tukey’s post hoc. n = 5–6 pups and 3–4 litters per time point and treatment group (total = 15 litters). No statistical differences were observed; n.s., not significant.

Proteins that are critical for intercellular communication and heart structure were assessed by qRT-PCR in whole-heart samples. No effect of treatment with LPS and/or hyperoxia was detected at, P0, P7, or P21 for connexin-43 mRNA expression. At P56, there was an interaction between sex and exposure, but no differences were observed within groups using post hoc analysis (Figure 7A). Likewise, there were no effects of exposure on desmin mRNA expression at P0, P7, or P56. At P21, there was an effect of exposure, with the Sal/RA-exposed group presenting higher expression than the LPS/RA group (Figure 7B).

## 4. Discussion

Fetal growth restriction and preterm birth are now well recognized as independent risk factors for the development of cardiac morbidities and early heart failure, with one epidemiological study reporting a relative risk of heart failure increased by 17 times in childhood and early adulthood for infants born at less than 28-week gestation [17,18,19,20]. Additionally, fetal growth restriction, a pathologic lack of reaching full fetal growth potential, affects between 3 and 7% of all newborns [21]. There is increasing epidemiologic evidence linking preterm birth and fetal growth restriction to cardiac pathologies later in life, including heart failure [22], cardiac hypertrophy [23], hypertension [24], and ischemic heart disease [25,26]. Clinical studies using echocardiography [18,19,27,28,29], cardiac magnetic resonance imaging [30], and tissue pathology [31] have revealed evidence of subclinical pathological cardiac indices associated with prematurity long before the onset of symptoms. Cardiac magnetic resonance imaging of adults who were born preterm demonstrates an abnormal phenotype with higher left and right ventricle mass and wall thickness independent of blood pressure, an altered three-dimensional structure, and decreased biventricular systolic function compared to adults who were born full-term [32]. Subtle abnormalities in heart structure and function have also been identified on echocardiograms of these patients prior to the development of clinical symptoms [27].

Our study revealed growth restriction, with lower postnatal body weights (Table 2) in the mice exposed to inflammation and/or hyperoxia, as we have previously reported. Notably, body weight was lower in mice exposed to LPS during intrauterine development from birth through P21, but at P21, there is a substantial increase in weight compared to the Sal/RA controls, indicative of weight compensation or respective overcompensation between P7 and 21. The physiological consequences of rapid postnatal catch-up growth are more significant than low birth weight alone for the development of cardiovascular disease in later life [33]. This observation is in line with epidemiological studies from Erikkson et al. reporting death from cardiovascular disease in men born with low birth weight but exhibiting an above average body mass index at all ages from 7 to 15 years [34]. Furthermore, at P56, females exhibited a significant lower body weight compared to the respective males, but there were no differences between treatments, implicating a sex-based protective effect. Interestingly, our echocardiographic calculations of LV mass index revealed the highest LV mass index in the male mice at P56 in the Sal/RA group (Figure 3). The male LPS/O_2_ group had the lowest LV mass index at P56, potentially representing disproportionately poor growth of the LV relative to the whole heart. This finding persisted even after normalizing LV mass to mouse body weight. Alternatively, we can speculate that hyperoxia exposure led to abnormal pulmonary vascular development and right ventricular hypertrophy as previously reported [35,36]. A low LV mass has also been observed in young adults born preterm, but no sex differences were identified [18].

Previous studies have shown important sex-related differences in cardiovascular outcomes in an older population [37]. We previously used our mouse model of inflammation and hyperoxia to evaluate cardiac function in 10-month-old mice [13]. LV function was analyzed using pressure–volume loops in vivo and revealed that male mice were more severely affected by adverse perinatal exposures and had worse contractile dysfunction than females [13]. The current study evaluated cardiac performance early in life to determine at what point abnormalities can be detected to facilitate early detection and interventions. Serial echocardiography revealed higher heart rates in the LPS/O_2_ group (Figure 1), likely representing a persistent adaptation of cardiac output in response to physiologic stress and increased cardiac output requirement during the perinatal period (Figure 2). Further, the LPS/O_2_ group exhibited LV systolic dysfunction, based on EF and SF measurements, in both sexes by P14, and persisting though P56 (Figure 2). A transient improvement was seen in the groups exposed to hyperoxia at P28, reflecting an adaptation after removal from hyperoxia at P14, but function subsequently worsened again by P56 relative to the Sal/RA groups (Figure 2), suggesting that the early hyperoxia exposure leads to irreversible functional changes that become evident even after initial recovery. Similar functional deficits have been observed in young adults born preterm with specific detriments to LV performance [19] and the risk of heart failure increased with decreasing gestational age at birth [20].

While we did not observe a clear pattern of echocardiographic changes prior to day 28, there are obvious adaptations occurring between P14 and P28 that result in more profound dysfunction by P56. The P14 to P28 ages are equivalent to adolescence in mice and the changes observed at these time points may be due to sexual maturity and the influence of sex hormones or further adaptations to early dysfunction as the mice mature [38]. By P56, which is a late adolescence or early adulthood time point, the cardiac deficiencies are evident and worsen with time, as previously observed in our model [12,13].

MHC, a key component of the sarcomere contraction, is developmentally regulated, with a key isoform switch from βMHC to αMHC predominance occurring in rodents within the first few days of postnatal life [39]. In the mouse, βMHC is the fetal isoform, which is characterized by lower adenosine 5′-triphosphate (ATP) utilization and higher endurance. The adult isoform is αMHC, which utilizes ATP at higher rates but also produces greater contractile strength [40]. The switch is carefully regulated, and alterations in the ratio of α- to βMHC are involved in the development of cardiac pathologies as a compensatory mechanism [40]. Reactivation of fetal gene expression is seen in heart failure, likely as a mechanism to conserve energy in a failing heart but at the expense of overall contractile function [41]. Relatively small increases in αMHC expression led to increased force generation; so, tight control of the αMHC to βMHC ratio is critical for maintaining normal heart function [42]. Upregulation of βMHC expression is also an early marker of cardiac hypertrophy [41]. In this study, the LPS-exposed females had lower αMHC mRNA and protein at P0 and P7 relative to the saline-exposed females (Figure 4 and Figure 5), and while these changes did not persist as the mice aged, they may be considered an early marker of dysfunction. This resulted in a lower αMHC-to-βMHC ratio at P0 and P7, but not at P21 or P56. This may represent a delayed switch in the MHC phenotype in the female mice, which may serve a protective role. Male mice exposed to LPS/O_2_ had normal expression of αMHC mRNA and protein compared to Sal/RA controls until P56. At P56, significantly higher αMHC levels were observed in males, resulting in a greater αMHC-to-βMHC ratios (Figure 4, Figure 5 and Figure 6). Disruptions in contractile proteins demonstrate a differential reaction to adverse exposures between males and females, suggesting long-term consequences in males consistent with sex-specific differences in cardiac morbidities and in vivo function [13,37]. Differences in the relative expression of αMHC to βMHC were also observed between mRNA and protein, indicating that post-transcriptional regulation is likely a contributing factor to differences between groups.

Desmin, an intermediate filament cytoskeletal protein, has been implicated in heart failure and many cardiomyopathies [43]. In this study, mRNA expression of desmin was not altered by exposure to inflammation and hyperoxia (Figure 7A). Previous studies using the LPS/O_2_ model have revealed alterations in desmin protein expression in older mice, which may represent a primary pathology or compensatory response [12,13]. Abnormal localization of connexin-43 protein, a key component of gap junctions, was also observed in previous studies following perinatal exposure to inflammation and hyperoxia. [12,13]. However, connexin-43 mRNA expression changes were not identified in this study at these early time points (Figure 7B).

The strengths of the current study are the serial echocardiograms performed in the same mice over the time period and the evaluation of the early time points for contractile protein changes. Weaknesses include the inability to perform reproducible echocardiography in mouse pups younger that P14 and insufficient tissues to perform Western blot analyses and immunohistochemistry on desmin and connexin-43 at the early time points. Nevertheless, this report further defines the importance of early exposures to adult cardiac pathologies.

## 5. Conclusions

In summary, the present study reveals that an adverse perinatal environment results in the abnormal development and function of the cardiac contractile apparatus. Our data further define differences between males and females in their responses and adaptations to these early-life insults. Molecular analyses confirm alterations in the expression of myosin heavy chain isoforms shortly after birth, and echocardiography demonstrates a physiological dysfunction, with males more severely affected than females. The pattern variability in cardiac function through early development would imply that mechanisms associated with compensatory weight gain may be in play early in life (P14–P28) but are not able to overcome the programming deviations caused by early exposures. We speculate that in humans, early life abnormalities in functional and molecular indices may represent cardiac remodeling and changes that partially explain the male proclivity towards long-term altered cardiac structure, function, and ultimately heart failure.

## Figures and Tables

**Table 1:** RT-PCR primer sequences.

Gene, Mouse	Sequence
β-actin	For: 5′-CCT GAC AGA CTA CCT CAT GAA GAT C-3′
	Rev: 5′-TAG AGC AAC ATA GCA CAG CTT CTC-3′
Connexin-43	For: 5′-ACA GCG GTT GAG TCA GCT TG-3′
	Rev: 5′-GAG AGA TGG GGA AGG ACT TGT-3′
Desmin	For: 5′-GTG GAT GCA GCC ACT CTA GC-3′
	Rev: 5′-TTA GCC GCG ATG GTC TCA TAC-3′
α-myosin heavy chain	For: 5′-GCC CAG TAC CTC CGA AAG TC-3′
	Rev: 5′-ATC AGG CAC GAA GCA CTC C-3′
β-myosin heavy chain	For: 5′-CCT GCG GAA GTC TGA GAA GG-3′
	Rev: 5′-CTC GGG ACA CGA TCT TGG C-3′

For, forward; rev, reverse.

**Table 2:** Mice body weights across age groups.

DOL	Sex	Sal/RA			Sal/O_2_			LPS/RA			LPS/O_2_			Two-Way ANOVA
	(Grams)	Mean	SEM	N	Mean	SEM	N	Mean	SEM	N	Mean	SEM	N	
P0	male	1.74	0.023	32				1.65 *	0.019	41				effect of sex, *p* = 0.001
	female	1.67	0.029	31				1.56 *	0.026	39				effect of exposure, *p* < 0.001
														
P7	male	5.03	0.099	42	4.35	0.143	36	4.74 *	0.083	45	4.33 *	0.112	24	effect of exposure, *p* < 0.001
	female	4.93	0.185	18	4.15	0.144	27	4.44 *	0.108	43	4.19 *	0.162	20	
														
P21	males	10.11	0.439	11	9.43	0.278	14	12.48 *^#^	0.746	13	11.00 *^#&^	0.576	14	effect of exposure, *p* < 0.001
	female	9.71	0.253	15	8.62	0.212	12	11.25 ^#^	0.639	10	9.97	0.437	17	interaction, *p* = 0.005
														
P56	males	24.03	0.260	3	24.52	0.490	6	27.61 *^#^	0.519	7	23.52 ^&^	0.686	5	effect of sex, *p* < 0.001
	female	21.37	0.888	7	19.55 ^	0.622	4	22.01 ^	0.698	5	19.00 ^	0.503	4	effect of exposure, *p* < 0.001

* different than Sal/RA; ^#^ different than Sal/O_2_; ^&^ different than LPS/RA; ^ different between sexes, same treatment.

## Data Availability

The datasets used and/or analyzed during the current study are available from the corresponding author on reasonable request.

## Supplementary Materials

The following supporting information can be downloaded at: https://www.mdpi.com/article/10.3390/jcdd11110346/s1, Table S1: LV volume, systole; Table S2: LV volume diastole; Table S3: LVPW thickness, systole; Table S4: LVPW thickness, diastole; Figure S1: M-mode images obtained from echocardiographs at P56.
