# Supplementary material for: Perinatal Inflammation Results in Sex-Dependent Cardiac Dysfunction
Source: J Cardiovasc Dev Dis. 2024 Nov 1;11(11):346. doi: 10.3390/jcdd11110346 (PMC11594672; doi:10.3390/jcdd11110346)

Supplemental Table S1. LV volume, systole

| DOL | sex (grams) | Sal/RA | | | Sal/O2 | | | LPS/RA | | | LPS/O2 | | | Two Way ANOVA |
|---|---|---|---|---|---|---|---|---|---|---|---|---|---|---|
| | | mean | SEM | N | mean | SEM | N | mean | SEM | N | mean | SEM | N | |
| P14 | male | 7.879 | 0.589 | 3 | 8.317 | 2.431 | 4 | 7.837 | 1.448 | 6 | 15.381 | 3.778 | 5 | effect of exposure p=0.042 |
| | female | 6.831 | 0.902 | 6 | 8.630 | 2.876 | 3 | 5.778 | 0.380 | 2 | 11.384 | 1.661 | 3 | |
| | | | | | | | | | | | | | | |
| P21 | male | 15.672 | 0.767 | 4 | 19.187 | 2.015 | 6 | 15.111 | 1.549 | 6 | 14.569 | 1.599 | 5 | n.s. |
| | female | 15.527 | 0.353 | 5 | 14.645 | 1.565 | 3 | 13.474 | 2.947 | 2 | 15.704 | 0.937 | 4 | |
| | | | | | | | | | | | | | | |
| P28 | males | 21.175 | 1.904 | 3 | 17.619 | 1.785 | 5 | 20811 | 1.567 | 6 | 17.519 | 0.732 | 5 | effect of sex p=0.012 |
| | female | 13.309 | 0.896 | 7 | 18.701 | 0.382 | 4 | 14.712 | 1.738 | 2 | 14.275 | 1.863 | 3 | interaction p=0.034 |
| | | | | | | | | | | | | | | |
| P56 | males | 24.564 | 2.386 | 3 | 30.821 | 3.331 | 6 | 35.688 | 2.975 | 6 | 23.116 | 2.542 | 4 | effect of sex p=0.005 |
| | female | 18.714 | 1.437 | 7 | 19.901 | 3.318 | 4 | 23.680 | 1.072 | 2 | 22.120 | 1.477 | 4 | |

Supplemental Table S2. LV volume, diastole.

| DOL | sex (grams) | Sal/RA | | | Sal/O2 | | | LPS/RA | | | LPS/O2 | | | Two Way ANOVA |
|---|---|---|---|---|---|---|---|---|---|---|---|---|---|---|
| | | mean | SEM | N | mean | SEM | N | mean | SEM | N | mean | SEM | N | |
| P14 | male | 25.820 | 2.354 | 3 | 23.718 | 2.370 | 4 | 27.327 | 4.058 | 6 | 38.258 | 6.556 | 5 | n.s. |
| | female | 22.215 | 1.477 | 6 | 25.567 | 7.407 | 3 | 18.838 | 0.615 | 2 | 30.647 | 6.765 | 3 | |
| | | | | | | | | | | | | | | |
| P21 | male | 39.620 | 2.044 | 4 | 44.117 | 3.820 | 6 | 43.496 | 3.984 | 6 | 39707 | 4.484 | 5 | n.s. |
| | female | 36.144 | 3.059 | 7 | 40.714 | 1.083 | 3 | 37.258 | 7.066 | 2 | 40.014 | 1.607 | 4 | |
| | | | | | | | | | | | | | | |
| P28 | males | 58.222 | 5.377 | 3 | 48.190 | 3.437 | 5 | 54.264 | 1.981 | 6 | 46.597 | 1.859 | 5 | effect of sex p=0.004 |
| | female | 42.740 | 1.175 | 7 | 47.624 | 1.242 | 4 | 46.776 | 3.524 | 2 | 37.451 | 4.006 | 4 | effect of exposure p=0.015 |
| | | | | | | | | | | | | | | |
| P56 | males | 70.150 | 3.523 | 3 | 68.668 | 4.624 | 6 | 73.187 | 4.493 | 6 | 49.508 | 3.222 | 4 | effect of sex p=0.003 |
| | female | 51.512 | 4.016 | 7 | 52.155 | 2.644 | 4 | 56.735 | 4.064 | 2 | 47.507 | 2.522 | 4 | effect of exposure p=0.010 |

Supplemental Table S3. LVPW thickness, systole.

| DOL | sex | Sal/RA | | | Sal/O2 | | | LPS/RA | | | LPS/O2 | | | Two Way ANOVA |
|---|---|---|---|---|---|---|---|---|---|---|---|---|---|---|
| | (grams) | mean | SEM | N | mean | SEM | | (grams) | mean | SEM | N | mean | SEM | |
| P14 | male | 1.082 | 0.065 | 3 | 1.024 | 0.087 | 5 | 1.250 | 0.23503 | 3 | 0.974 | 0.355 | 2 | n.s. |
| | female | 0.980 | 0.213 | 6 | 0.987 | 0.117 | 4 | 1.105 | 0.330 | 2 | 0.0946 | 0.199 | 3 | |
| | | | | | | | | | | | | | | |
| P21 | male | 1.011 | 0.259 | 3 | 1.161 | 0.148 | 6 | 1.395 | 0.175 | 6 | 0.822 | 0.153 | 5 | n.s. |
| | female | 1.004 | 0.113 | 7 | 1.072 | 0.202 | 4 | 0.955 | 0.190 | 4 | 1.196 | 0.395 | 4 | |
| | | | | | | | | | | | | | | |
| P28 | males | 0.649 | 0.162 | 3 | 1.163 | 0.182 | 5 | 0.905 | 0.136 | 3 | 0.984 | 0.284 | 2 | n.s. |
| | female | 1.027 | 0.162 | 7 | 0.986 | 0.146 | 4 | 0.670 | 0.357 | 2 | 1.133 | 0.301 | 3 | |
| | | | | | | | | | | | | | | |
| P56 | males | 1.575 | 0.447 | 3 | 1.307 | 0.138 | 6 | 1.157 | 0.1270 | 6 | 1.124 | 0.198 | 5 | n.s. |
| | female | 0.954 | 0.123 | 7 | 1.183 | 0.119 | 4 | 0.884 | 0.334 | 2 | 1.560 | 0.170 | 4 | |

Supplemental Table S3. LVPW thickness, diastole.

| DOL | sex | Sal/RA | | | Sal/O2 | | | LPS/RA | | | LPS/O2 | | | Two Way ANOVA |
|---|---|---|---|---|---|---|---|---|---|---|---|---|---|---|
| | (grams) | mean | SEM | N | mean | SEM | N | mean | SEM | N | | mean | SEM | |
| P14 | male | 0.670 | 0.076 | 4 | 0.691 | 0.082 | 5 | 0.898 | 0.053 | 3 | 0.695 | 0.360 | 2 | n.s. |
| | female | 0.710 | 0.177 | 6 | 0.688 | 0.156 | 4 | 0.599 | 0.225 | 2 | 0.776 | 0.198 | 3 | |
| | | | | | | | | | | | | | | |
| P21 | male | 0.800 | 0.249 | 4 | 0.902 | 0.150 | 6 | 1.022 | 0.180 | 6 | 0.604 | 0.057 | 5 | n.s. |
| | female | 0.888 | 0.143 | 6 | 0.811 | 0.323 | 4 | 0.953 | 0.192 | 2 | 0.966 | 0.325 | 4 | |
| | | | | | | | | | | | | | | |
| P28 | males | 0.300 | 0.078 | 4 | 0.698 | 0.150 | 5 | 0.655 | 0.209 | 2 | 0.568 | 0.208 | 2 | n.s. |
| | female | 0.662 | 0.164 | 7 | 0.672 | 0.216 | 4 | 0.670 | 0.357 | 2 | 0.730 | 0.307 | 3 | |
| | | | | | | | | | | | | | | |
| P56 | males | 0.963 | 0.257 | 4 | 0.927 | 0.146 | 6 | 0.856 | 0.148 | 6 | 0.733 | 0.157 | 5 | n.s. |
| | female | 0.608 | 0.126 | 7 | 0.672 | 0.119 | 4 | 0.884 | 0.334 | 2 | 1.219 | 0.180 | 4 | |

Supplemental Figure S1. M-mode images obtained from echocardiographs at P56.

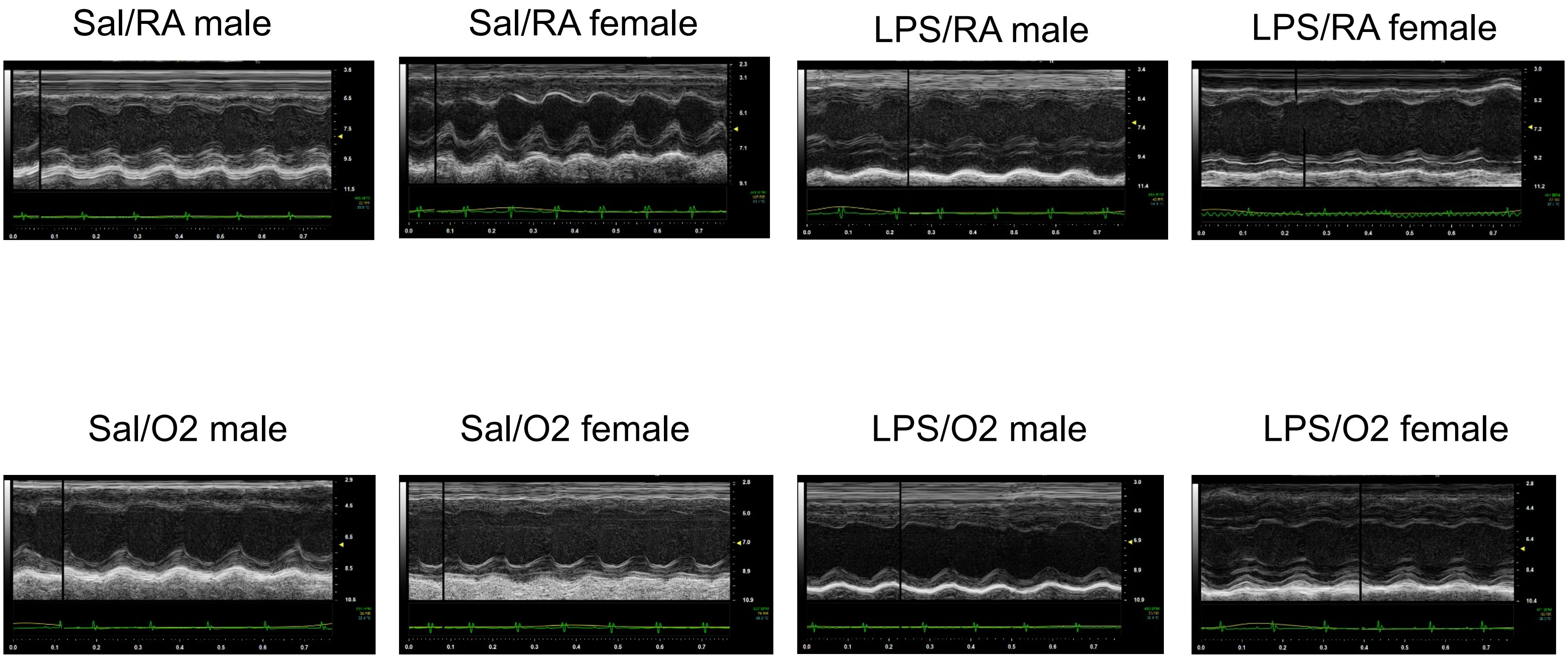

Supplement: Supplementary file 1 [file jcdd-11-00346-s001.zip › jcdd-3183704-supplementary.pdf]
